# Mothering While Sick: Poor Maternal Health and the Educational Attainment of Young Adults

**DOI:** 10.1177/00221465241247538

**Published:** 2024-04-29

**Authors:** Shannon Cavanagh, Athena Owirodu, Lindsay Bing

**Affiliations:** 1 University of Texas, Austin, TX, USA; 2 University of North Carolina, Chapel Hill, NC, USA; 3 Columbia University, New York, NY, USA

**Keywords:** educational attainment, maternal health, mothering, transition to adulthood

## Abstract

At a time when educational attainment in young adulthood forecasts long-term trajectories of economic mobility, better health, and stable partnership, there is more pressure on mothers to provide labor and support to advance their children’s interests in the K–12 system. As a result, poor health among mothers when children are growing up may interfere with how far they progress educationally. Applying life course theory to the National Longitudinal Study of Adolescent to Adult Health to investigate this possibility, we found that young adults were less likely to graduate from college when raised by mothers in poor health, especially when those mothers had a college degree themselves. Young people’s school-related behaviors mediated this longitudinal association. These findings extend the literature on the connection between education and health into an intergenerational process, speaking to a pressing public health issue—rising morbidity among adults in midlife—and the reproduction of inequality within families.

After decades of economic restructuring and globalization, young people across sociodemographic lines are increasingly expected to complete postsecondary degrees as they transition into adulthood. A college degree is now more consequential in terms of socioeconomic security, and it also shapes nonsocioeconomic domains like health, longevity, and family formation across adulthood (Goldin and Katz 2008; Hout 2012). Consequently, pressures on parents to get their children into and through college have dramatically increased, raising expectations for parenting behaviors that actively support, control, and advance young people’s path through the K–12 system to higher education. This pressure has been greatest for mothers (Hays 1996; Lareau 2011).

Reflecting such gendered pressures, ample research has detailed the distinct ways that mothers are encouraged to cultivate skills and orientations in their children that increase their chances for higher education; manage extracurricular activity participation, coursework, college counseling, and other aspects of education now deemed crucial to college-going; handle socioemotional challenges of adolescent development that pose academic risks; and maintain a visible presence at school (e.g., meeting with teachers, volunteering) to signal their families’ investment in students’ success (Calarco 2014, 2018; Crosnoe 2011; Lewis and Diamond 2015). Any factor that can interfere with mothers’ academic socialization of their children and their management of children’s educational pursuits, therefore, has the potential to disrupt their children’s eventual educational attainment in young adulthood.

This study focuses on poor maternal health as one such risk factor. Rising morbidity and mortality rates among U.S. adults in midlife (Case and Deaton 2015) suggest that a significant number of adults are simultaneously raising children and managing acute and chronic health issues. Building on research that considers parents’ health in early childhood (e.g., Frech and Kimbro 2011) and children’s own health (e.g., Hardie and Turney 2017), we contend that exploring the association between maternal health and their children’s educational trajectories is also important. Just as children who feel bad have trouble learning and achieving (Crosnoe, Bonazzo, and Wu 2015), mothers who feel bad may have trouble navigating their teens’ movement through the academic and social hurdles of secondary schooling in a social context in which they are expected to be in charge. Moreover, their health problems might keep them from attaining or capitalizing on the kinds of resources (e.g., money, networks) that promote adolescents’ academic prospects in the neoliberal educational system of the United States and both distract and trouble their adolescents in ways that interfere with their full engagement in school (Hogan, Shandra, and Msall 2007). Drawing on a conceptual model derived from life course theory, we tested this possibility through analyses of data on mothers and adolescents from the National Longitudinal Study of Adolescent to Adult Health (Add Health) and its linked transcript data from the Adolescent Health and Academic Achievement (AHAA) study.

## Background

### Changes in Mothers’ Health and Parenting Expectations

This study seeks to combine two significant trends in the United States. The first concerns rising levels of poor health (and mortality) among adults in midlife, including women (Bound et al. 2015; Case and Deaton 2015). The second concerns the increasing demands and expectations around mothering as a reaction to the new reality of a global economy (Schmidt et al. 2023). Connecting these two trends can shed valuable light on the life chances of contemporary youth, proxied by their educational attainment in young adulthood.

Starting with adult heath, U.S. life expectancy has stalled since 2010 (Mehta, Abrams, and Myrskylä 2020), with much slower improvements in life expectancy compared to its peer nations since at least 1990 (Crimmins et al. 2011). This disparity (and more recent decline) in U.S. life expectancy is likely tied to rising levels of hypertension, diabetes, lung disease, obesity, cancer, suicide, drug use, and chronic pain among U.S. adults (Bound et al. 2015; Case, Deaton, and Stone 2020). Collectively, these conditions translate into not only a lower life expectancy overall but also worse health and increasing chronic conditions among adults in early midlife (Bound et al. 2015; Case et al. 2020). Rising morbidity at midlife may be an important mechanism for the intergenerational transmission of status because it is occurring at a stage in the life course when many are rearing teenagers and also managing their careers and supporting their parents (i.e., the sandwich generation; see Lachman, Teshale, and Agrigoroaei 2015). It has been established that poor health makes performing work more difficult (Collins et al. 2005), and this can be extended to raising and launching teenagers.

At the same time, parenting styles have shifted such that mothers are increasingly engaged in intensive mothering as a reaction to global economic transformations marked by increasing labor market competition, economic uncertainty, and other macro-level disruptions (e.g., the Great Recession; Cooper 2014). The ideology of intensive parenting argues that mothers are better parents than fathers and, among other things, are the most appropriate person to ensure their child’s successful development and future success, a future that is determined, in part, by their academic abilities (Dow 2015; Lareau and Weininger 2003). This kind of mothering can start very soon after a child is born (Budds et al. 2017; Randles 2021) and remains important throughout adolescence and the transition to adulthood (Norris and van Hasselt 2023). Here, we focus on mothering adolescents through the middle school and high school years. Mothering during these years happens at a critical translation point between early life experiences and future social and economic trajectories (Johnson, Crosnoe, and Elder 2011).

### Health in Maternal Generation and Educational Careers in the Child Generation

These two trends—worsening health in midlife and increasing social expectations on mothers—are important in their own right and so too is the connection between them. Following insights from life course theory and status attainment model, this study connects these trends by viewing them both as part of the linked lives phenomenon, where experiences and statuses in the maternal life course trickle down to the child’s life course through parenting and parental involvement (Blau and Duncan 1967; Elder 1998). More specifically, the intergenerational transmission of status, including educational attainment, is tied to the ways parents provide supports, experiences, and resources to their children and also interact with schools and other institutions on their child’s behalf. This labor, in turn, supports their children and signals to the child and to their educators that academic success and college-going is prioritized and expected.

How might the linked lives phenomenon operate when a mother is in poor health? Consistent with Turney and Hardie (2018), we consider health as a resource like money or time that is needed to perform one’s role as a parent. Thus, mothers in good health may be better able to engage in the parenting behaviors tied to adolescent academic success, such as daily discussions about classes or homework, that can keep children engaged academically in middle school and high school (Hill and Tyson 2009) because they have more energy and fewer health-related limitations. With good health, they may also have greater capacity to support teens as they navigate the emotional highs and lows of adolescence, such as negotiating romantic relationships and friendship dynamics (Crosnoe 2011), making them more interested in staying in high school and going to college. Conversely, mothers in poor health may have less time and energy to invest in their children through parenting. Managing a chronic health condition, feeling unwell or unhappy, or being restricted by a disability may reduce a mother’s capacity to support their adolescents as they navigate academic hurdles associated with advanced course taking in high school and weather the socioemotional challenges that can define adolescence and derail their educational careers. Poor maternal health can also shape family processes within the home, introducing stress between parents and disrupting the flow of time, attention, and supervision to children (Frech and Kimbro 2011; Hardie and Landale 2013; Hogan et al. 2007; Turney and Hardie 2018).

Differences in parenting expectations and behaviors tied to maternal health may also be related to adolescent behaviors in school and out. With extra strain and limited attention at home, young people may seek out other sources of support and status with peers (Hogan et al. 2007), caring less about the future and engaging in more problem behaviors (Jørgensen et al. 2022). Having a mother in poor health may also dampen adolescents’ desire to attend college because they expect to forgo or delay college in favor of earning an income to support their families or feel like they cannot be away from home (Bratti and Mendola 2014).

Still, mothers who feel bad might actually become more involved in their children’s education lives. Although little work has considered the ways maternal health might shape parental involvement, scholarship on mothers who are low-income (Fong 2020) or have histories of incarceration (Branigan et al. 2023; Gurusami 2019) suggest that women with contested or precarious statuses as mothers often engage in compensatory parenting behaviors, especially as it relates to their children’s educational lives. For example, Branigan et al. (2023) find that maternal incarceration is associated with more school-based care relative to mothers who did not have histories of incarceration. Formerly incarcerated mothers also engaged more directly in home-based school behaviors with their kids, often as a way to “demonstrate themselves as engaged and responsible mothers” to their children (Branigan et al. 2023). Although the stigma associated with incarceration is likely more significant than the stigma associated with poor health, it is possible that mothers in poor health might become motivated to engage in school-related behaviors that can minimize the value of good health as a source of capital, translating into a null or even a positive association between poor health and children’s educational status in young adulthood.

For these reasons, the first aim of this study is to examine the association between maternal health status and young people’s educational status during the transition to adulthood.

### Mediation and Moderation of Linked Maternal Health and Child Education

Once we establish the linkage between mother’s health and young people’s educational status, the next step is to identify mechanisms that underlie it. Previously, we highlighted how the school-related behaviors and expectations of mothers and teens may play a role. These behaviors and expectations are guided by life course theory’s view of educational status in young adulthood as part of a young person’s social pathway of education (Crosnoe and Johnson 2011). Whether a young person drops out or stops at high school, attends college, or graduates with a bachelor’s degree is determined, in part, by earlier school-related experiences that can be shaped by their maternal parenting and vary by her health status. Thus, the second aim of the study considers young people’s early assessment of their mothers’ involvement and expectations about schooling and their own behaviors and dispositions at the start of high school.

To be sure, children undergo important cognitive growth during adolescence and begin to see themselves as autonomous, efficacious individuals. They are better able to anticipate the consequences of their actions, learn from their success and failures, and coordinate the pursuit of their goals, academic and otherwise (Steinberg 2005). As such, they are often better able to make decisions about course taking and coursework and school-based behaviors and begin to see how their choices matter for their future in a way that is different from childhood (Eccles, Wigfield, and Byrnes 2003). Still, maternal involvement remains important to young people’s educational success in middle and high school (Norris and van Hasselt 2023). For these reasons, we consider factors that capture young people’s perspective of their mothers’ expectations and supports for their education and their own behaviors, academic and otherwise, during early adolescence as potential mechanisms linking maternal health and how far they go in school.

We start with measures that capture young people’s view of their mothers’ expectations and behaviors around schooling, all captured during early adolescence. First, we consider young people’s more abstract assessment of “academic socialization,” or a set of strategies that mothers provide to communicate their expectations for their children’s educational careers, foster their educational aspirations, and help them plan for the future (Hill and Tyson 2009). Collectively, this kind of labor captures expectations and behaviors that guide young people as they navigate the increasingly cumulative coursework demands in high school and can determine how far they go in school. Mothers’ health can make this kind of labor harder or easier to do.

We also consider young people’s own behaviors in school. First, we consider math coursework at the start of high school. Math sets the stage for later academic success in advanced courses and scoring well on standardized tests (Long, Conger, and Iatarola 2012). Just as those who begin by taking more advanced courses and earning higher grades have greater opportunities to excel academically across high school, those who begin high school earning low grades and taking lower-level courses have few opportunities to recover and excel over time (Riegle-Crumb 2006). We also consider school absence (Gottfried and Hutt 2019), school suspensions (Morris and Perry 2016), and young people’s reports about trouble in the classroom. Collectively, these behaviors capture young people’s presence and performance in school in early adolescence that can be shaped by maternal inputs, including health.

Life course theory also emphasizes that linked lives can play out differently in the context of a stratified, unequal society that gives some more access to opportunities and others fewer opportunities and fewer buffers when setbacks emerge. These differences produce and maintain inequality (Bourdieu 1984). Intensive mothering was originally viewed as a predominantly White, middle-class-based strategy for transferring advantage to their children (Blair-Loy 2003), and ethnographic work on educational trajectories highlighted clear social class differences in parenting (Calarco 2014; Chin and Phillips 2004; Lareau 2011). Still, elevated expectations of mothers, especially how they engage with institutions, exist across the socioeconomic spectrum and racial-ethnic lines (Collins 2022; Dow 2015; Elliott, Powell, and Brenton 2015). Notably, the bind of intensive mothering might be even more pernicious for women with limited economic and social means to meet these expectations. Higher socioeconomic status (SES) mothers—especially White mothers—may be better able to translate these practices into advantage for their adolescents, but all mothers operate under largely unreachable expectations when it comes to launching their children (Hays 1996). For these reasons, we use mothers’ own education as a marker of the family’s position in the stratified system of U.S. education and expect it might condition the link between maternal health and young people’s educational status in young adulthood.

To be sure, maternal education is linked with both health status and mothering strategies. The stall in life expectancy, especially for women, is largely driven by significant increases in poor health among women without college degrees (Sasson 2016). At the same time, mothers with college degrees often have a greater sense of belonging in schools (Khan 2012) and understand better how schools, as institutions, operate. They are often better able to deploy and pay for supports for their teens and to signal to teachers and administrators their expectations for their children (Calarco 2018). They are also more likely to be married (Smock and Schwartz 2020), which can translate to more parenting support for teens and greater access to economic resources and health insurance to absorb the costs associated with poor health. Mothers with lower levels of education may be less comfortable in schools and have fewer resources to offer to schools and to spend in ways that increase their teen’s chances of more schooling (Bourdieu 1984). These mothers are also more likely to be unpartnered (Smock and Schwartz 2020) or primary earners (Glass, Raley, and Pepin 2021) relative to mothers with a college degree, meaning they may have less time to invest in their children’s schooling (Ressler et al. 2017).

Our third aim, then, is to explore the moderating role of maternal education in this linked lives phenomenon by testing two competing hypotheses. One is that mothers’ poor health represents a double disadvantage by exacerbating existing disadvantages associated with lower maternal education, which would lead to a stronger association between maternal health and young people’s educational status among those whose mothers have less formal education. The other is that poor health for college-educated mothers will be more consequential for their children’s educational trajectories because poor health may degrade their ability to transfer their advantage through the intensive parenting expected of them.

## Data And Methods

### Analytic Sample

The data for this research came from the National Longitudinal Study of Adolescent to Adult Health (Add Health), a nationally representative study of adolescents in Grades 7 through 12 in the United States in 1995 (Harris 2012). Add Health used a multistage, stratified, school-based cluster sampling design. For each study school, Add Health collected an in-school survey from every student (about 90,000) who attended on the day of administration. About one year later, Add Health selected a nationally representative sample from this pool of students to participate in the Wave I in-home interview, conducted between April and December 1995. Parents of adolescents who participated in the in-home interview completed a separate survey instrument at this time (n = 17,670). The Wave IV interview, conducted in 2008, captured respondents ages 24 through 32 and included Wave I high school seniors (n = 15,701). Approximately 73% of the original Wave I sample completed the Wave IV questionnaire. Other waves (Waves II, III, V) have been collected but were not used in this study.

We also used data from the Adolescent Health and Achievement Study (AHAA), a transcript study, that supplemented Add Health with detailed information about educational careers (see Muller et al. 2007). During Wave III data collection, respondents were asked to complete a high school transcript release form that authorized the study personnel to collect transcripts from the last schools the respondents attended. Approximately 91% of the Wave III respondents signed a valid transcript release form, and transcript data were obtained for approximately 12,000 participants. These data provide official grades, indicators of course-taking patterns at the student and school levels, and educational contexts within and among schools that can be linked to the data from the Add Health survey.

The analytic sample for this study was limited to young people who completed Waves I and IV and had valid Wave IV sample weights and whose biological mothers completed the parent questionnaire. We restricted the sample to biological mothers because chronic health condition indicators that we used to construct the maternal health scale were collected for biological mothers only. More than 87% of the sample retained in Wave IV had parent questionnaires completed in Wave I by biological mothers. We observed varying levels of missingness in our analytic sample across the different sources of data: 4% of the sample were missing data on mothers’ self-reported health and chronic conditions, 31% had no academic transcript data from the AHAA study, and fewer than 2% were missing data on adolescent-reported school measures (ever suspended, have trouble in school, desire for college) from the Wave I interview. We used multiple imputation in Stata 18 to create imputed data sets, following the methods described in Enders (2022). For all analyses, including descriptive statistics, we pooled estimates and standard errors from the imputed data sets. Our analytic sample, adjusted with cross-sectional Wave IV survey weights, included 10,698 young people.

### Measures

#### Educational status in young adulthood

At Wave IV, young people reported the highest grade or degree they earned in school, with choices ranging from eighth grade or less up to completed a doctoral degree. These responses were coded as less than high school, high school diploma or GED, some college but no four-year degree, and at least a bachelor’s degree. As shown in the sample summary statistics presented in Table 1, 7% had not completed high school, 19% had only a high school diploma, 42% had completed some college, and 31% had earned a bachelor’s degree or higher by Wave IV. Approximately 16% of respondents were attending postsecondary education at the time of the survey, and 28% of respondents had not yet finished a bachelor’s degree but reported that they expected to do so in their lifetimes. As such, our estimates were not capturing lifetime educational attainment but educational status by at least 26, the age minimum for 99% of the sample.

**Table 1.** Weighted Summary Statistics for Key Analytic Variables (*N* = 10,698).

	Mean/Percentage	SE	Minimum	Maximum	Health Index Weight
Educational status in young adulthood					
No high school degree	7.00				
High school degree/GED	19.22				
Some college	42.31				
At least a bachelor’s degree	31.47				
Individual characteristics					
Respondent’s sex (1 = female)	49.70				
Age at Wave 4	28.75	1.75	23.00	35.00	
Race-ethnicity					
White	69.00				
Black	14.16				
Asian American	2.79				
Native American	2.00				
Latine	11.88				
Other	.16				
Maternal characteristics					
Scale of poor maternal health	.00	.02	−1.00	8.00	
Self-reported health:					
Overall physical health (5 = poor, 1 = excellent)	2.37	1.02	1.00	5.00	.05
Disability status	5.64				.11
Obesity	19.87				.13
Migraines	28.03				.12
Allergies	43.12				.13
Asthma	9.45				.08
Alcoholic	1.96				.12
Diabetes	5.21				.11
Unhappy	3.97				.14
Maternal age	4.97	5.54	21.00	69.00	
Maternal educational attainment					
No high school degree	15.90				
High school degree/GED	32.92				
Some college	29.17				
At least a college degree	22.01				
Family structure					
Two bio parents	61.18				
Mother and stepparent	15.31				
Single mother	23.52				
Any welfare receipt	9.70				
Academic socialization at Wave 1					
Desire to attend college	4.41	.03	1.00	5.00	
Sense of maternal disappointment about not going to college	3.93	.04	1.00	5.00	
Sense of maternal involvement in school	1.65	.02	.00	4.00	
Early academic behaviors in school					
GPA in math courses	2.20	.03	.00	4.00	
On track for college (Algebra I by Grade 9)	77.58				
Self-reported scale of academic trouble in school	2.11	.02	1.00	5.00	
Ever suspended	25.37				
Excused absences in past year					
0	9.54				
1–2	29.33				
3–10	46.50				
10+	14.63				

*Source*: National Longitudinal Study of Adolescent to Adult Health, Waves I and IV and the Adolescent Health and Academic Achievement Study.

#### Poor maternal health

At Wave I, mothers answered a battery of questions about their physical and mental well-being. First, they rated their physical health on a scale of 1 (excellent) to 5 (poor). This measure is considered a valid indicator of health and is predictive of overall mortality risk (Benyamini and Idler 1999; Ferraro and Farmer 1999). Mothers also reported on whether they had any of the following health conditions: diabetes, asthma, obesity, alcoholism, allergies, migraines. They also reported whether they were happy or not or were disabled. These nine items were correlated such that mothers who reported being in fair or poor health were significantly more likely to report each of the health conditions. To maximize our measure of maternal health and increase efficiency by minimizing the number of correlated predictors included in our model, we created a summary index using the *swindex* command in Stata 18 (Schwab et al. 2021). The *swindex* command is a generalized least squares weighting procedure proposed by Anderson (2008) that applies greater weight to the least well correlated items, thus maximizing new information captured by the item (the weights associated with each item are included in Table 1). The scale ranged from –1 to 8, with a mean of 0 and standard deviation of 1; higher scores indicated worse maternal health. About 14% of mothers reported having health 1 SD below the mean.

#### Mediators

We included two sets of measures that might capture the ways that maternal health is linked with young people’s educational status in young adulthood. The first set of potential mediators captured young people’s academic socialization in early adolescence. The first item gauged how disappointed young people think their mother would be if they did not earn a bachelor’s degree, and the second measured how much they want to go to college. Both items were measured on a 5-point scale, with higher values associated with higher levels of academic socialization in the family. We also included a scale of maternal involvement in education, ranging from 0 to 3, that indicated whether in the past four weeks their mother talked with them about schoolwork or grades, helped them work on a project for school, or talked about other things that they were doing in school.

The second set of measures got at young people’s behaviors in school. Academic performance was based on math course taking variables collected in the AHAA transcript study. We used respondent’s math grade in Grade 9 and whether they were enrolled in or had already completed Algebra 1 at Grade 9, a measure of being “on time” for math or in a position to complete the high school math sequence expected for college (Riegle-Crumb 2006). To get at their behavior in school, we included a binary measure of self-reported suspension (1 = ever suspended, 0 = never suspended) and a scale measuring the mean responses to self-reported academic and classroom struggles, including having trouble completing homework, getting along with teachers, and paying attention at school in the past week (1 = never, 5 = every day). We also included self-reported absence in the current academic year. All mediators were measured at Wave 1.

#### Moderator

Mothers’ education was based on mothers’ reports of how far they went in school, ranging from no formal schooling up through professional training beyond a four-year college degree. These categories were coded into four dummy variables: less than high school, high school or GED (reference category), some college, and bachelor’s degree or higher.

#### Covariates

We included several control variables to account for selection into poor maternal health and young people’s education status in young adulthood. Starting with mothers, we included their self-reported age in whole years, self-reported receipt of welfare, and family structure at Wave I using a three-category variable (married or cohabiting with child’s biological father, married or cohabiting with another partner, single). Young people’s characteristics included self-reported biological sex, race and ethnicity, and age at Wave IV.

### Analytical Approach

To understand the association between maternal health status during adolescence and later educational status, we estimated bivariate correlations among key analytic variables. We then estimated a series of nested ordered logistic regression models given that the outcome was measured as an ordinal variable. Model 1 estimated the focal association between the maternal health scale and educational status in young adulthood net of the covariates that may predict both maternal health status and young people’s educational attainment. Model 2 added measures of young people’s academic socialization and course taking and behaviors in school to explore their potential to mediate the focal association. Model 3 included an interaction between maternal health and maternal educational attainment to explore the degree to which the focal association varied by social class. To ease interpretation of ordinal logistic regression coefficients, we present the average marginal effect of poor maternal health on predicted educational status, which we estimated using the *mimrgns* command in Stata 18 (Klein 2014).

Finally, we recognized that the link between maternal health and later educational status could be due to unobserved variables. To address this possibility, we tested the sensitivity of our results. We calculated the impact threshold for confounding variables by using the *Konfound-it* test package in R to quantify how much bias there would have to be due to omitted variables to invalidate any inferences we made (see Frank et al. 2013). We present these sensitivity analyses in the following.

## Results

### Descriptive Results

Table 2 presents the bivariate association between maternal reports of poor health and other variables, including a set of health conditions reported for biological mothers at Wave I that might underlie these reports. About 14% of respondents had a mother whose health was more than 1 SD worse than the mean and are considered in poor health for these descriptive analyses. Across the board, mothers in poor health reported being in worse physical health overall and more chronic health conditions, including obesity, migraines, diabetes, asthma, and having allergies, than did those in good health. They were also more likely to report being alcoholic, unhappy, and disabled. These mothers were also less likely to be college graduates and more likely to be single parents than mothers who reported better health. They were twice as likely to receive welfare at Wave I compared with other mothers. Turning to young people’s own characteristics, those with mothers in poor health were more likely to be Black and Native American.

**Table 2.** Maternal Health at Wave 1 by Key Analytic Measures (*N* = 10,698).

	In Poor Health (>1 SD above Mean) (*n* = 1,521)	Not in Poor Health (<1 SD above) (*n* = 9,177)
Educational status in young adulthood		
No high school degree	11.27	6.27
High school degree/GED	22.51	18.66
Some college	43.50	42.10
At least a bachelor’s degree	22.72	32.97
Individual characteristics		
Respondent’s sex (1 = female)	50.08	49.63
Age at Wave 4	28.89 (1.81)	28.73 (1.74)
Race-ethnicity		
White	64.48	69.77
Black	17.00	13.68
Asian American	1.77	2.96
Native American	4.27	1.62
Latine	12.23	11.82
Other	.25	.15
Maternal characteristics		
Self-reported health		
Overall physical health (5 = poor, 1 = excellent)	3.15 (1.10)	2.23 (.96)
Disability status	23.37	2.56
Obesity	51.15	14.60
Migraines	60.62	22.52
Allergies	73.21	38.08
Asthma	37.37	4.74
Alcoholic	11.23	.38
Diabetes	25.51	1.85
Unhappy	21.62	1.01
Maternal age	41.09 (5.61)	40.95 (5.53)
Maternal educational attainment		
No high school degree	21.23	15.00
High school degree/GED	30.63	33.31
Some college	32.07	28.67
At least a college degree	16.07	23.02
Family structure		
Two bio parents	49.46	63.18
Mother and stepparent	15.25	15.32
Single mother	35.29	21.51
Any welfare receipt	19.46	8.03
Academic socialization at Wave 1		
Desire to attend college	4.39 (1.11)	4.42 (1.04)
Sense of maternal disappointment about not going to college	3.83 (1.31)	3.95 (1.24)
Sense of maternal involvement in school	1.50 (1.13)	1.67 (1.13)
Early academic behaviors in school		
GPA in math courses	2.01 (.98)	2.23 (.97)
On track for college (Algebra I by Grade 9)	73.73	78.25
Self-reported scale of academic trouble in school	2.21 (.89)	2.09 (.84)
Ever suspended	33.63	23.96
Excused absences in past year		
0	7.71	9.85
1–2	22.65	30.47
3–10	48.85	46.10
10+	20.79	13.58

*Source*: National Longitudinal Study of Adolescent to Adult Health, Waves I and IV and the Adolescent Health and Academic Achievement Study.

Importantly, young people with mothers in poor health were nearly twice as likely to not finish high school and much less likely to complete a bachelor’s degree than those with a mother in better health. As for academic socialization, those with mothers in poor health reported that their mothers would be less disappointed if they did not attend college and were less involved in their schooling. These young people were also less likely to have completed at least Algebra 1 by the end of ninth grade, had lower math grades, were more likely to be suspended, had trouble with school, and missed 10 or more days of school than did other young people.

Taken together, these results suggest that maternal health may be meaningfully linked with young people’s educational attainment and that young people’s early experiences in school may explain this link. The multivariate analyses described next explored whether these bivariate associations remained significant once covariates were taken into account.

### Linked Lives of Mothers and Their Children

**Figure 1.**
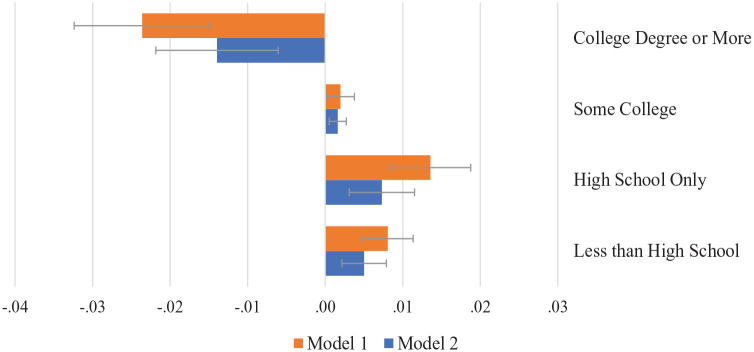
Average Marginal Effect of Poor Maternal Health on Educational Attainment in Young Adulthood. *Source*: National Longitudinal Study of Adolescent to Adult Health, Waves I and IV and the Adolescent Health and Academic Achievement Study.

Results from ordinal logistic regression models are shown in Table 3. Model 1 tested the first aim, exploring the association between maternal health and educational status at Wave IV, net of key covariates. Echoing bivariate results, young people with mothers who reported worse health were significantly less likely to go further in formal schooling than other youth. In terms of average marginal effects, displayed in Figure 1, findings from this model show that for every standard deviation increase in poor maternal health, the predicted probability of a young person completing college decreases by an average of approximately 2.4 percentage points, and the probability that they go no further than high school increases by close to 1.4 percentage points. In other words, young people with sick mothers are significantly less likely than their peers to go to college or complete a college degree.

**Table 3.** Ordered Logistic Regression Coefficients for Models Exploring Link between Maternal Health and Later Educational Status (*N* = 10,698).

	Model 1		Model 2		Model 3	
	Coefficient	SE	Coefficient	SE	Coefficient	SE
Maternal poor health scale	−.13***	(.03)	−.09***	(0.03)	−.01	(.05)
Individual characteristics						
Female	.55***	(.06)	.27***	−.06	.27***	(.06)
Race and ethnicity (White = reference)						
Black	−.14	(.12)	.11	(.11)	.11	(.11)
Asian American	.34	(.26)	.13	(.20)	.11	(.20)
Native American	−.36	(.23)	−.18	(.23)	−.19	(.23)
Latine	−.03	(.10)	.11	(.10)	.10	(.10)
Other	−.71+	(.37)	−.68	(.66)	−.63	(.67)
Age at Wave 4	−.01	(.03)	.06	(.03)	.06*	(.03)
Maternal characteristics						
Maternal age	0.04***	(.01)	.03***	(.01)	.03***	(.01)
Maternal education (high school/GED)						
No high school degree	−.84***	(.10)	−.61***	(.11)	−.61***	(.11)
Some college	.56***	(.07)	.39***	(.07)	.4***	(.07)
At least a college degree	1.62***	(.10)	1.17***	(.09)	1.16***	(.09)
Family structure at Wave 1 (two biological parents = reference)						
Mother and stepparent	−.52***	(.08)	−.36***	(.08)	−.36***	(.08)
Single mother	−.55***	(.06)	−.26***	(.07)	−.26***	(.07)
Any welfare receipt	−.55***	(.11)	−.48***	(.12)	−.50***	(.13)
Academic Socialization at Wave 1						
Desire to attend college (not at all = reference)						
Not so much			.23	(.27)	.23	(.27)
Moderately			.63**	(.21)	.62**	(.21)
Very much			.76***	(.20)	.76***	(.20)
Extremely			1.43***	(.19)	1.42***	(.19)
Mother would be disappointed if you didn’t go to college (not at all = reference)						
Not so much			.43**	(.16)	.43**	(.16)
Moderately			.44**	(.15)	.44**	(.15)
Very much			.70***	(.14)	.70***	(.14)
Extremely			.59***	(.14)	.59***	(.14)
Scale of mothers’ involvement in school activities			.07*	(.03)	.07*	(.03)
Early academic orientation and behaviors in school						
On track for college (Algebra I by Grade 9)			.86***	(.04)	.86***	(.04)
Math GPA			.45***	(.08)	.45***	(.08)
Academic troubles at school—self-reported at Wave 1			−.01	(.04)	−.01	(.04)
Ever suspended—self-reported at Wave 1			−.45***	(.07)	−.45***	(.07)
School absences						
1–2			.02	(.10)	.02	(.10)
3–9			.04	(.10)	.04	(.10)
≥10			−.41***	(.12)	−.41***	(.12)
Maternal health × Maternal education						
Poor health × No high school degree					−.09	(.08)
Poor health × Some college					−.12+	(.07)
Poor health × At least a college degree					−.21*	(.09)
Cutoff for high school graduate only	−1.45+	(.73)	3.49***	(.78)	3.46***	(.78)
Cutoff for some college	.31	(.72)	5.56***	(.77)	5.53***	(.77)
Cutoff of at least a college graduate	2.54***	(.71)	8.31***	(.77)	8.29***	(.77)
Observations	10,698		10,698		10,698	

*Source*: National Longitudinal Study of Adolescent to Adult Health, Waves I and IV and the Adolescent Health and Academic Achievement Study.

+ p < .01 *p < .05 **p < .01 ***p < .001.

Turning to measures that proxy social location, results from Model 1 show that maternal education, welfare receipt, and family structure were significantly associated with young people’s educational attainment. Young people with mothers who earned more education were more likely to go further in schooling than all others, and those who received welfare or resided outside of a two-biological parent household were significantly less likely to go further in school net of all other factors.

Model 2 tested the second aim, exploring whether the aforementioned focal association was explained by academic socialization in the home and school-related behaviors in early adolescence. Not surprisingly, each indicator that tapped academic socialization was significantly associated with educational attainment in young adulthood. For example, young people who reported that their mother engaged more school-related activities with them or would be more disappointed if they did not go to college at Wave I were significantly more likely to report higher educational attainment in young adulthood. Similarly, young people who reported higher college aspirations at Wave I reported higher educational attainment in young adulthood. Measures of young people’s behaviors in schools also were associated with eventual educational attainment. For example, being on time in the math course-taking sequence and earning a higher GPA were positively associated with later educational attainment. Self-reported school suspension at Wave I and reporting more than 10 days of excused absences were negatively associated with educational attainment net of other factors.

Importantly, the coefficient associated with the poor maternal health index was attenuated by about 30% when measures of school behaviors were added to the model (separate models not shown) but remained significantly associated with educational attainment in young adulthood. Figure 1 illustrates the average marginal effect of poor maternal health estimated from Models 1 and 2 for each of the four levels of educational attainment. After controlling for school behaviors and academic socialization with Model 2, each standard deviation increase in poor maternal health was associated with a 1.4 percentage point decrease in the probability of completing a college degree. These findings suggest that part of the association between maternal health and educational status operates through young people’s behaviors in school at the start of high school. Yet even considering these factors, maternal health continued to matter for how far young people went in school by age 26. As expected, measures of academic socialization were associated with educational attainment, yet they did little to change the link between maternal health and educational attainment.

### Socioeconomic Status, Maternal Health, and Educational Attainment

**Figure 2.**
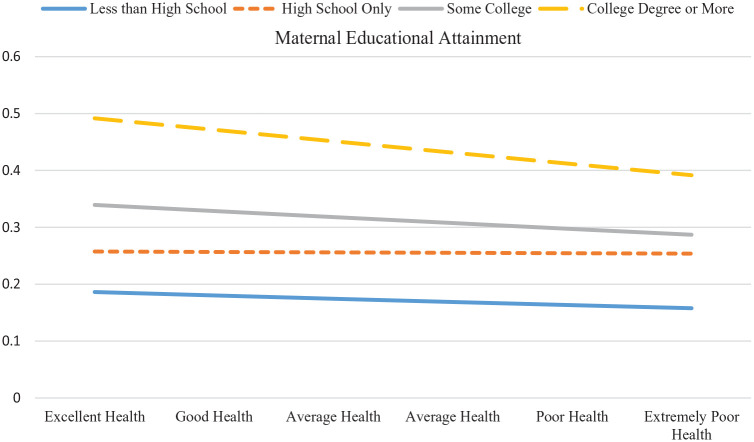
Average Marginal Effects of Poor Maternal Health on the Predicted Probability of Young People Completing College (N = 10,698). *Source*: National Longitudinal Study of Adolescent to Adult Health, Waves I and IV and the Adolescent Health and Academic Achievement Study.

Model 3 tested the third aim, interacting maternal education with maternal health. Here, we considered whether maternal education—a proxy for social class (McLanahan 2004)—conditioned the effect of maternal health status on young people’s educational attainment. The interaction terms reached statistical significance, which suggests that having a mother in poor health mattered most for young people whose mothers had at least a college degree. For these young people, having a mother in poor health led to significantly lower educational attainment by Wave IV. Figure 2 illustrates the average marginal effect of poor maternal health on the predicted probability of completing a college degree by maternal education level. For young people whose mothers had a college degree, each standard deviation increase in poor maternal health is associated with a 3.9 percentage point decline in the likelihood that they complete a college degree themselves, but it has almost no effect for young people whose mothers had only a high school degree. Poor maternal health also decreased the probability of more education for young people whose mothers had less than a high school degree and only some college, but these effects did not reach statistical significance.

### Sensitivity Analysis

Finally, we checked the robustness of our results for the possibility of omitted variable bias using the *Konfound-it* package in R (www.konfound-it.org). After pooling estimates from Model 2, the fully specified model, we used this procedure to quantify how much bias there would have to be due to omitted variables or any other source to invalidate our inference. This analysis indicated that 34.7% of the estimate effect of maternal health on young people’s educational status in young adulthood would have to be due to bias to invalidate the observed association. In other words, invalidating the inference would require replacing more than one-third of the observations with null hypothesis cases of no effect for maternal health not to matter net of the controls and mediators included.

## Discussion

In the context of neoliberal economic and social transformations, a college degree is viewed as a necessary condition for a “good” life. Consequently, parenting norms have shifted in ways that are expected to facilitate its pursuit (Calarco 2018; Hays 1996; Lareau 2011). These changing norms, consistent with the ideology of intensive parenting, view mothers and mothering as central to this task, with mothers, mostly, organizing young people’s lives in ways that support them as they navigate academic hurdles and socioemotional challenges and maintaining a visible presence at schools to signal their investment in their students’ success (Calarco 2018). Importantly, these shifts are happening at the same time as the health of American adults, including women, has grown worse, with increasing chronic conditions emerging earlier in the life course (Bound et al. 2015; Montez et al. 2011). Taken together, this suggests that many women are engaging in or are expected to engage in more intensive parenting and managing their own poor health.

Using life course theory and status attainment model as a guide, we considered how shifts in parenting and maternal health played out in terms of young people’s educational status in young adulthood, if this association was explained by other school-related behaviors, and for whom this association was strongest. Doing so can highlight the cascading effects of poor adult health on children’s life chances and point to the need to strengthen school and family partnerships in middle and high school in the context of chronic health conditions for those who parent children.

Two central findings emerged, speaking to the intergenerational transmission of inequality in ways that call for responses from the health care and educational systems. The first is that maternal health was strongly associated with how far young people went in school in young adulthood. Maternal health was associated with young people’s behaviors in school, and these factors predicted educational attainment, partially mediating the focal association between maternal health and educational status at young adulthood. Still, the association between maternal health and educational attainment remained significant. The second is that this association between mother’s health and child’s educational attainment appears to vary by SES; having a mother in poor health was the most detrimental for those whose mothers had the most advantage. Children of these mothers were significantly less likely to go to college when their mothers reported being in poor health when they were in early adolescence than others. These findings raise three insights about health, parenting, and social mobility.

First, theories about mothering rarely consider health status as a factor that might complicate women’s household and emotional labor for children and teenagers. Meaningful differences in health emerge in early midlife, just as many women are engaging in intensive parenting practices, and as these data collected in the early 1990s demonstrate, appear to matter to their children’s life chances. This pattern is likely much stronger today when the adult population is less healthy overall. As such, just as scholars foreground women’s union status or educational attainment when thinking about the economic, social, and psychological resources that they draw on to parent and adapt to changing demands, we should do the same for women’s health. Doing so can illuminate unmeasured heterogeneity among families.

Second, young people’s reports of maternal academic socialization were associated with how far young people went in school but did not mediate the link between maternal health and young people’s educational attainment. This suggests that mothers, regardless of health status, protect that part of their work as a parent. Instead, maternal health may shape more general household strategies and practices (e.g., bed times, curfews) that, in turn, matter to young people’s schooling. Future work that includes interviews with families where a mother is less healthy can help illuminate the pathways that link maternal health with children’s school-related behaviors. It can also demonstrate the ways that mothers in poor health “make it work,” especially in the absence of standardized paid sick leave and universal health care in the United States, which can make managing poor health and mothering even more difficult. Future work should also consider how the health of fathers and other adults in the home matter to young people’s educational careers and how a sick father might “distract” mothers from managing their children’s development.

Third, these findings say something about the way social class operates in the United States. Children of the most advantaged mothers (those with a college degree) experienced the sharpest change in their (unequal) likelihood of completing college in the face of poor maternal health. To be sure, these mothers were less likely to be in poor health, and their children were more likely to earn more education in young adulthood, on average, but when mothers reported poor health, the educational trajectories of their children were the most disrupted.

Why might children of more advantaged mothers be more disrupted? This may be because these young people and their families are embedded in schools and communities where resources are more plentiful but “opportunity hoarding” is more prevalent (Lewis and Diamond 2015; Sattin-Bajaj and Roda 2020). As such, when more advantaged mothers feel bad, they may be less able to do the work needed to garner opportunities and supports for their children, opportunities and supports that can keep young people engaged in school and on track to graduate from college. Less advantaged mothers, on the other hand, have fewer resources to leverage for their children, making poor maternal health less consequential for their children’s educational trajectories. Alternatively, this association might be methodological, with more vulnerable families, including those more likely to have unhealthy mothers and college-going young people, underrepresented in longitudinal, school-based studies like Add Health, making the comparison groups less valid (Pettit 2012).

Still, that poor maternal health is more consequential for more advantaged children is consistent with other family scholarship that suggests that family structure change, for example, is more detrimental to children born in two married parent families versus those born in cohabiting or single parent families (Bzostek and Berger 2017; Ryan and Claessens 2013) or for those living in higher income households compared to those in lower income homes (Bloome 2017; Ryan, Claessens, and Markowitz 2015). Collectively, this work points to the precarity of class position even among the more advantaged. Poor health or a divorce in the adult generation, for example, can have a cascading effect on the class position of the child generation.

Before building on this research to explore these possibilities, some limitations need to be addressed. First, we are relying on a scale of maternal health, one that includes only a crude indicator of mental health, at a single point in time. Health status is dynamic, and developing more nuanced and dynamic measures of maternal health trajectories can deepen our understanding of the interplay between maternal health and their children’s educational trajectories. Still, a robust literature finds that self-rated health is a good indicator of overall health (Ferraro and Farmer 1999; Idler and Benyamini 1997), suggesting that our findings are a good starting point on which to build. A second limitation is our lack of measurement of mothering, of the time and activities that mothers do as part of the management of their children’s schooling. We are relying on children’s reports of mothers’ school-related behaviors and expectations, not mothers’ reports. Although these indirect indicators—some from adolescent reports, some from school transcripts—give a handle on the adolescent’s movement through school, measures of household parenting behaviors can give greater insight into how the linked lives of mother and adolescent unfold in sickness and in health.

Moving forward, we will explore these linkages with longitudinal data sets like ECLS-K:2011, a prospective cohort that follows children from kindergarten through fifth grade, capturing a more recent cohort of parents—mothers and fathers—and children over time, better establishing early patterns of parenting behaviors that can set the stage of later academic attainment. We will also explore these linkages with the PSID-CDS study and Transition into Adulthood Supplements, which includes detailed measures of health and educational trajectories, children’s and family time use, and parenting measures, following mothers, fathers, and their children across the childhood and the transition to adulthood. The pursuit of such avenues—by us or others—is important because it deepens our understanding of the ways health shapes parenting and the intergenerational transmission of dis/advantage. It also illuminates another way that institutions like schools and health systems can come together to support teens whose parents are in poor health.
